# Event and Comment

**Published:** 1914-03-15

**Authors:** 


### DENTAL REGISTER.

**Vol. LXVIII. March 15, 1914. No. 3**

### EVENT AND COMMENT.

"SOME THINGS TO BE CONSIDERED." Under this title the Editor of the New Jersey Dental Journal writes in the February issue of the present status of the dental profession in general terms, and closes his observations with the following specific statement of the future lines of progress or endeavor:

"If dentistry is to occupy the position which is opening to it, the work of investigation and research must be continued with unabated energy and enthusiasm; furthermore, every dentist must advance in scientific knowledge if we are to attain or maintain this higher standing. Our colleges will have to give their students more thorough courses of study and instruction, and our State Boards of Dental Examiners must raise the standards of requirements for license to practice." The editorial further says these are matters of *vital interest* and should be given careful thought for they concern the future of dentistry.

Now we are wondering if this is just a nice piece of writing, or whether the editor thought this statement through so carefully that he really means what he has written. In other words is there a crisis impending, and has our editor a vision of the promised land? In the first placd is there an important work for our profession today that it has not always faced with a somewhat clear view, although at times the way out of the overshadowing mist was a bit obscured? It is true that we are just now in the stimulated atmosphere of prevention as opposed to relief and repair of former ages, and possibly our knowledge of somethings is more definite, but are we sufficiently settled in our convictions that we can enter upon what will undoubtedly be a campaign of instruction before a conquest can be even predicted?

The Editor has indicated the plan of campaign. The colleges must give more complete courses of instruction and must increase the quality. If our Campaigner is a college teacher he will know that there has been an increase at least in quantity of dental instruction in the past twenty years of at least one hundred per cent., while it is the general belief of those who are watching this matter most closely, that the quality of instruction was never better, if so good as it is today. If he will consult the admission requirements, as printed in the proceedings of the National Faculties Association for the year 1887, he will find that all that was required at that time was a Grammar or Eighth Grade School education for admission to our Dental Schools. At present the requirements are a four year high school education. If he consult the curiculum requirement of the same period he will find that we are today teaching more than double the number of subjects by more than double the number of teachers and with facilities in the way of building and equipment that were not then even seen in dreams. Besides this, today many of our stronger schools have teachers who are giving their entire time to instruction and research work, where twenty years ago no such condition was found anywhere.

Our Editor also says our State Boards must be more severe with their test, that only prepared men shall be licensed. Twenty years ago our State Laws were ineffective in intent as well as in fact. It is true that they are not up to our ideals today, but they are wonderfully more efficient and much stronger than at that period and they are being constantly revised and improved so that our laws and State Board Methods are as nearly up to the standards of our professional attainment and popular sentiment as it is practicable to make them.

Now we do not wish to appear critical, or to be understood as claiming full attainment of our ideals, or to be satisfied with present conditions. We want to look at this matter in the largest way possible, but we would like to know if there is to any large extent a sentiment in the profession that can be depended on for support of such a forward movement as our Editor friend suggests. Does he know what it will mean to carry out his ideas? Has he counted the cost? If the colleges are to increase standards, more money, much more, must be provided. Many schools would today be insolvent were it not for the heroic sacrifices of loyal instructors who care more for their professions welfare, than for their own financial intereste. How can research workers be provided without money? Will our State Laws and more rigid examinations interfere with the present demand for the privilege of utilizing partially trained office assistants to put into effect the propaganda for clean teeth? We see the vision of our confrere and have seen it for many days, but we must confess that its comprehension staggers our optimism, and we are therefore asking how it can be done. We see it and we want it, but we are wondering if the professions see it enough to make the sacrifices to obtain it. If as our Editor suggests every dentist would become more scientific, this would undoubtedly help some, but does he not know that at least three fourths of the members of our profession do not even read the Current Literature, and how can they know what is transpiring, or how can they be expected to have an interest in the scientific advancement of our profession?

DENTAL DISPENSARY RECORD. The November issue of this valued journal closes its interesting career of four years of devotion to the cause of oral hygiene. The editor is not tired out, but he can't find a business manager who will stick to the job, and the editor has some personal obligations to his family that claim at least a portion of his time and energy, and so he has concluded that the cause for which he has so strenuously labored is sufficiently well organized and entrenched in the minds of the profession generally that there is no longer a necessity for this publication. Now we are extremely sorry to have this most worthy and capable influence withdrawn just at a time when it seems to us most needed. The real work of oral hygiene has only just begun. A good deal of beating the air has been done and it had to be done to start the profession to thinking, but the real work of organization and accomplishment is yet to be done, and it will need the help of such men as Dr. Belcher and his sharp editorial pen to set this movement going and keep up its momentum until the habit becomes so fixed that he can honorably quit the field of battle and know that the campaign will continue to a successful issue. It is really too bad, so good a cause could not command the money and time of those who are capable and whose devotion to the cause would ensure its ultimate success. We know that Belcher and the entire profession of Rochester has backed up and kept this journal in the field and it does seem strange that it could not have continued its good work; as it had a sphere of its own that was much needed.

We shall miss the literary feast prepared by Belcher and Line, as there is always something worth while in their writings, and they have a literary style that puts prosaic material into interesting garb, so that it always attracts the eye as well as tickles the mentality. We trust that the habit of literary work shall not lapse with either, now that the pressure is released, but that other channels for reaching the profession with their stirring messages shall open to them.

